# Supplementary material for: A subclass of evening cells promotes the switch from arousal to sleep at dusk
Source: bioRxiv. 2023 Aug 29:2023.08.28.555147. Preprint. [Version 1] doi: 10.1101/2023.08.28.555147 (PMC10491161; doi:10.1101/2023.08.28.555147)
Supplement: Supplement 1 [file NIHPP2023.08.28.555147v1-supplement-1.pdf]

bioRxiv preprint doi: https://doi.org/10.1101/2023.08.28.555147; this version posted August 29, 2023. The copyright holder for this preprint (which was not certified by peer review) is the author/funder, who has granted bioRxiv a license to display the preprint in perpetuity. It is made available under aCC-BY 4.0 International license.

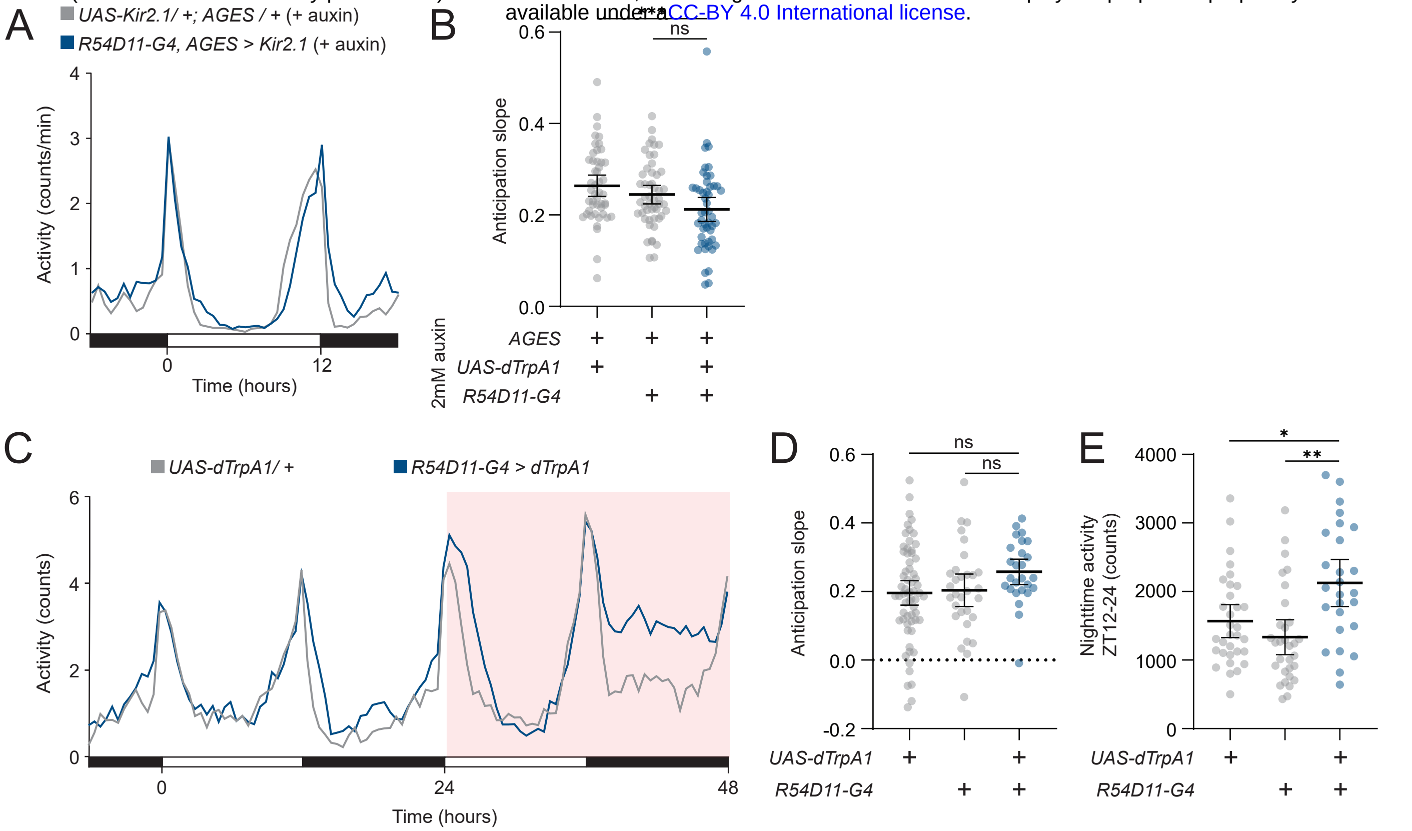

Figure S1

bioRxiv preprint doi: https://doi.org/10.1101/2023.08.28.555147; this version posted August 29, 2023. The copyright holder for this preprint (which was not certified by peer review) is the author/funder, who has granted bioRxiv a license to display the preprint in perpetuity. It is made available under aCC-BY 4.0 International license.

Figure S1 – Altering the activity of E2 cells does not significantly affect evening anticipation, Related to Figure 1

(A) Conditional electrical silencing of E2 neurons. Activity counts per min profile for 24 hrs for *R54D11-GAL4, AGES>UAS-Kir2.1* (blue) and *UAS-Kir2.1/+; AGES/+* (gray) flies on 2 mM NAA sucrose agar in 12:12 LD plotted in 30 min bins.

(B) Slope of evening anticipation for E2 conditional silencing. Average slope of evening anticipation (see Methods) plotted for *R54D11-GAL4, AGES>UAS-Kir2.1* (blue, n=49), *R54D11-GAL4, AGES/+* (gray, n=51), and *UAS-Kir2.1/+; AGES/+* (gray, n=48) flies. Mean ± 95% confidence interval is shown. One-way ANOVA with Bonferroni post hoc test.

(C) E2 neuron conditional activation. Activity counts per min profile for 48 hrs for *R54D11-GAL4>UAS-dTrpA1* (blue) and *UAS-dTrpA1* (gray) flies in 12:12 LD plotted in 30 min bins. Red background indicates increased temperature (28°C), compared to 22°C baseline.

(D) Slope of evening anticipation for E2 conditional activation. Average slope of evening anticipation (see Methods) plotted for activation day for *R54D11-GAL4>UAS-dTrpA1* (blue, n=26), *R54D11-GAL4/+* (gray, n=31), and *UAS-dTrpA1* (gray, n=32) flies. Mean ± 95% confidence interval is shown. One-way ANOVA with Bonferroni post hoc test.

(E) Nighttime activity with E2 conditional activation. Average number of activity counts at night (ZT12-24) for *R54D11-GAL4>UAS-dTrpA1* flies (blue, n=26), *R54D11-GAL4/+* (gray, n=31), and *UAS-dTrpA1* (gray, n=32) flies. Mean ± 95% confidence interval is shown. One-way ANOVA with Bonferroni post hoc test.

bioRxiv preprint doi: https://doi.org/10.1101/2023.08.28.555147; this version posted August 29, 2023. The copyright holder for this preprint (which was not certified by peer review) is the author/funder, who has granted bioRxiv a license to display the preprint in perpetuity. It is made available under aCC-BY 4.0 International license.

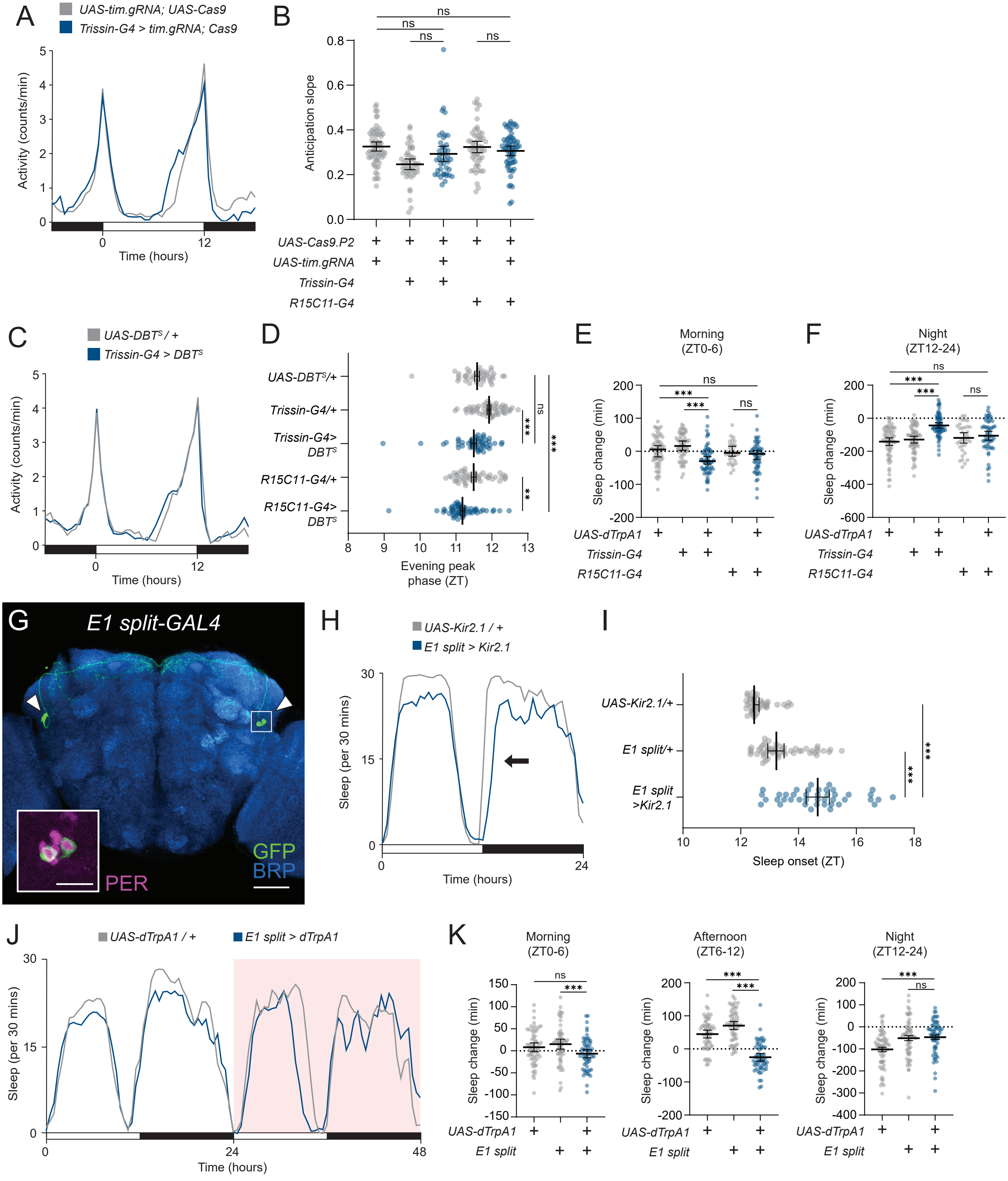

Figure S2

bioRxiv preprint doi: https://doi.org/10.1101/2023.08.28.555147; this version posted August 29, 2023. The copyright holder for this preprint (which was not certified by peer review) is the author/funder, who has granted bioRxiv a license to display the preprint in perpetuity. It is made available under aCC-BY 4.0 International license.

Figure S2 – Electrical activity, not core clock function, in E1 neurons alters behavior around dusk, Related to Figure 2

(A) Tissue-specific knockout of *timeless* specifically in E1 neurons. Activity counts per min profile for 24 hrs for *Trissin-GAL4>UAS-tim.gRNA, UAS-Cas9.P2* (blue) and *UAS-tim.gRNA/+; UAS-Cas9.P2/+* (gray) flies in 12:12 LD plotted in 30 min bins.

(B) Slope of evening anticipation for E1 neuron *tim* knockout. Average slope of evening anticipation (see Methods) for *Trissin-GAL4>UAS- tim.gRNA, UAS-Cas9.P2* (blue, n=43), *R15C11-GAL4>UAS- tim.gRNA, UAS-Cas9.P2* (blue, n=61), *Trissin-GAL4>UAS-Cas9.P2* (gray, n=46), *R15C11-GAL4>UAS-Cas9.P2* (gray, n=54), and *UAS-tim.gRNA/+; UAS-Cas9.P2/+* (gray, n=64) flies. One-way ANOVA with Bonferroni post hoc test.

(C) DBT^{S} expression specifically in E1 neurons. Activity counts per min profile for 24 hrs for *Trissin-GAL4>UAS-DBT*^{S} (blue) and *UAS-DBT*^{S}/+ (gray) flies in 12:12 LD plotted in 30 min bins.

(D) Phase of evening anticipation for E1 neuron DBT^{S} expression. Average phase of evening anticipation for *Trissin-GAL4>UAS-DBT*^{S} (blue, n=51), *R15C11-GAL4>UAS-DBT*^{S} (blue, n=60), *Trissin-GAL4/+* (gray, n=57), *R15C11-GAL4/+* (gray, n=49), and *UAS-DBT*^{S}/+ (gray, n=41) flies. One-way ANOVA with Bonferroni post hoc test.

(E) Morning sleep change with E1 neuron activation. Change in morning (ZT0-6) sleep amount with thermogenetic activation compared to baseline afternoon in *Trissin-GAL4>UAS-dTrpA1* (blue, n=63), *R15C11-GAL4>UAS-dTrpA1* (blue, n=55), *Trissin-GAL4/+* (gray, n=63), *R15C11-GAL4/+* (gray, n=34), and *UAS-dTrpA1/+* (gray, n=66). Same flies as Figs. 2D-E. Mean ± 95% confidence interval is shown. One-way ANOVA with Bonferroni post hoc test.

(F) Nighttime sleep change with E1 neuron activation. Change in nighttime (ZT12-24) sleep amount with thermogenetic activation compared to baseline afternoon in *Trissin-GAL4>UAS-*

bioRxiv preprint doi: https://doi.org/10.1101/2023.08.28.555147; this version posted August 29, 2023. The copyright holder for this preprint (which was not certified by peer review) is the author/funder, who has granted bioRxiv a license to display the preprint in perpetuity. It is made available under aCC-BY 4.0 International license.

dTrpA1 (blue, n=63), *R15C11-GAL4>UAS-dTrpA1* (blue, n=55), *Trissin-GAL4/+* (gray, n=63), *R15C11-GAL4/+* (gray, n=34), and *UAS-dTrpA1/+* (gray, n=66) flies. Same flies as Figs. 2D-E. Mean ± 95% confidence interval is shown. One-way ANOVA with Bonferroni post hoc test.

(G) Immunostaining of E1 split-GAL4 driver. Whole-mount immunostaining of an *R18H11-AD; R78G02-DBD* brain expressing *UAS-CD8::GFP* with anti-GFP (green), anti-BRP (blue), and anti-PER (magenta, inset) antibodies. E1 cells indicated by arrowheads. PER staining highlighted in solid white inset (LNds). Scale bars, 50 µm (main) and 15 µm (inset).

(H) E1 split-GAL4 silencing behavioral trace. Sleep amount per 30 min (right) profiles for 24 hrs of *R18H11-AD; R78G02-DBD>UAS-Kir2.1* (blue) and *UAS-Kir2.1/+* (gray) flies in 12:12 LD plotted in 30 min bins. Arrow indicates delay in sleep onset.

(I) Time of sleep onset with E1 split-GAL4 electrical silencing. Sleep onset time plotted by ZT for *R18H11-AD; R78G02-DBD>UAS-Kir2.1* (blue, n=42), *R18H11-AD; R78G02-DBD /+* (gray, n=51), and *UAS-Kir2.1/+* (gray, n=41) flies. Median ± 95% confidence interval is shown. Kruskal-Wallis with Dunn's post hoc test.

(J) E1 split-GAL4 24 hr thermogenetic activation in LD. Sleep profile for 48 hrs for *R18H11-AD; R78G02-DBD>UAS-dTrpA1* (blue) and *UAS-dTrpA1/+* (gray) flies in 12:12 LD plotted in 30 min bins. Red background indicates increased temperature (28°C), compared to 22°C baseline.

(K) Daily sleep change with E1 split-GAL4 activation. Change in morning (ZT0-6, left), afternoon (ZT6-12, middle), and nighttime (ZT12-24, right) sleep amount with thermogenetic activation compared to baseline afternoon in *R18H11-AD; R78G02-DBD>UAS-dTrpA1* (blue, n=58), *R18H11-AD; R78G02-DBD/+* (gray, n=59), and *UAS-dTrpA1/+* (gray, n=61) flies. Mean ± 95% confidence interval is shown. One-way ANOVA with Bonferroni post hoc test.

bioRxiv preprint doi: https://doi.org/10.1101/2023.08.28.555147; this version posted August 29, 2023. The copyright holder for this preprint (which was not certified by peer review) is the author/funder, who has granted bioRxiv a license to display the preprint in perpetuity. It is made available under aCC-BY 4.0 International license.

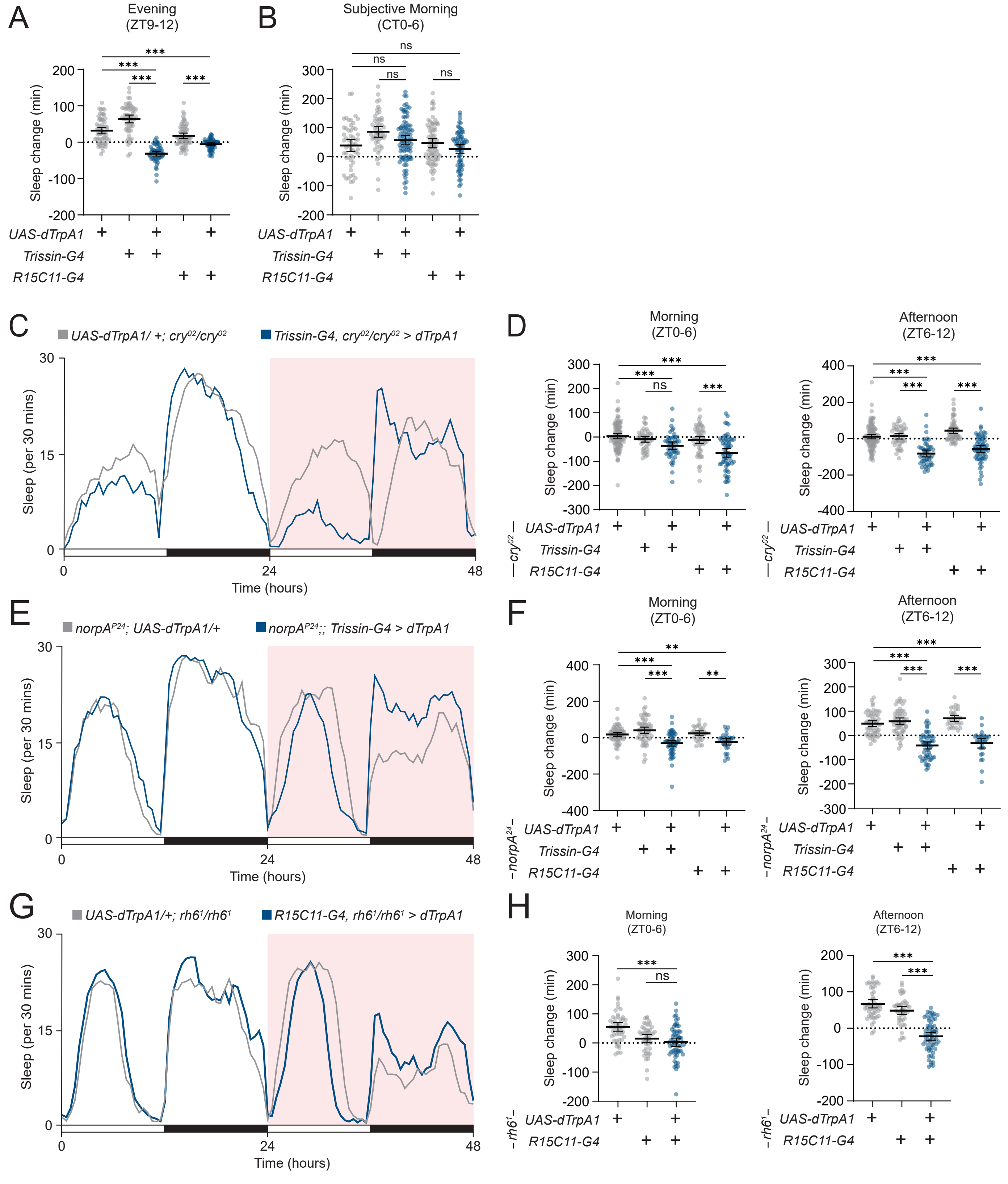

Figure S3

bioRxiv preprint doi: https://doi.org/10.1101/2023.08.28.555147; this version posted August 29, 2023. The copyright holder for this preprint (which was not certified by peer review) is the author/funder, who has granted bioRxiv a license to display the preprint in perpetuity. It is made available under aCC-BY 4.0 International license.

Figure S3 – Light-dependent output of E1 neuron activation depends on parallel pathways, Related to Figure 3

(A) Evening sleep change with E1 neuron activation. Change in evening (ZT9-12) sleep amount with thermogenetic activation compared to baseline afternoon in *Trissin-GAL4>UAS-dTrpA1* (blue, n=51), *R15C11-GAL4>UAS-dTrpA1* (blue, n=58), *Trissin-GAL4/+* (gray, n=56), *R15C11-GAL4/+* (gray, n=63), and *UAS-dTrpA1/+* (gray, n=59) flies. Same flies as Figs. 3A-B. Mean ± 95% confidence interval is shown. One-way ANOVA with Bonferroni post hoc test.

(B) Subjective morning sleep change with E1 neuron activation in DD. Change in morning (CT0-6) sleep amount with thermogenetic activation compared to baseline afternoon in *Trissin-GAL4>UAS-dTrpA1* (blue, n=88), *R15C11-GAL4>UAS-dTrpA1* (blue, n=68), *Trissin-GAL4/+* (gray, n=52), *R15C11-GAL4/+* (gray, n=74), and *UAS-dTrpA1/+* (gray, n=54) flies. Same flies as Figs. 3C-D. Mean ± 95% confidence interval is shown. One-way ANOVA with Bonferroni post hoc test.

(C) E1 neuron 24 hr thermogenetic activation in LD in *cry* mutants. Sleep profile for 48 hrs for *Trissin-GAL4, cry*^{02}*>UAS-dTrpA1; cry*^{02} (blue) and *UAS-dTrpA1/+* (gray) flies in 12:12 LD plotted in 30 min bins. Red background indicates increased temperature (28°C), compared to 22°C baseline.

(D) Daytime sleep change with E1 neuron activation in *cry* mutants. Change in morning (ZT0-6, left) and afternoon (ZT6-12, right) sleep amount with thermogenetic activation compared to baseline afternoon in *Trissin-GAL4, cry*^{02}*>UAS-dTrpA1; cry*^{02} (blue, n=47), *R15C11-GAL4, cry*^{02}*>UAS-dTrpA1; cry*^{02} (blue, n=63), *Trissin-GAL4, cry*^{02}*/cry*^{02} (gray, n=47), *R15C11-GAL4, cry*^{02}*/cry*^{02} (gray, n=60), and *UAS-dTrpA1/+; cry*^{02}*/cry*^{02} (gray, n=109) flies. Mean ± 95% confidence interval is shown. One-way ANOVA with Bonferroni post hoc test.

bioRxiv preprint doi: https://doi.org/10.1101/2023.08.28.555147; this version posted August 29, 2023. The copyright holder for this preprint (which was not certified by peer review) is the author/funder, who has granted bioRxiv a license to display the preprint in perpetuity. It is made available under aCC-BY 4.0 International license.

(E) E1 neuron 24 hr thermogenetic activation in LD in *norpA* mutants. Sleep profile for 48 hrs for *norpA*^{P24}*;; Trissin-GAL4>UAS-dTrpA1* (blue) and *norpA*^{P24}*; UAS-dTrpA1/+* (gray) flies in 12:12 LD plotted in 30 min bins. Red background indicates increased temperature (28°C), compared to 22°C baseline.

(F) Daytime sleep change with E1 neuron activation in *norpA* mutants. Change in morning (ZT0-6, left) and afternoon (ZT6-12, right) sleep amount with thermogenetic activation compared to baseline afternoon in *norpA*^{P24}*;; Trissin-GAL4>UAS-dTrpA1* (blue, n=55), *norpA*^{P24}*;; R15C11-GAL4>UAS-dTrpA1* (blue, n=30), *norpA*^{P24}*;; Trissin-GAL4/+* (gray, n=58), *norpA*^{P24}*;; R15C11-GAL4/+* (gray, n=31), and *norpA*^{P24}*; UAS-dTrpA1/+* (gray, n=57) flies. Mean ± 95% confidence interval is shown. One-way ANOVA with Bonferroni post hoc test.

(G) E1 neuron 24 hr thermogenetic activation in LD in *rh6* mutants. Sleep profile for 48 hrs for *R15C11-GAL4, rh6*^{1}*>UAS-dTrpA1; rh6*^{1} (blue) and *UAS-dTrpA1/+; rh6*^{1}*/rh6*^{1} (gray) flies in 12:12 LD plotted in 30 min bins. Red background indicates increased temperature (28°C), compared to 22°C baseline.

(H) Daytime sleep change with E1 neuron activation in *rh6* mutants. Change in morning (ZT0-6, left) and afternoon (ZT6-12, right) sleep amount with thermogenetic activation compared to baseline afternoon in *R15C11-GAL4, rh6*^{1}*>UAS-dTrpA1; rh6*^{1} (blue, n=62), *R15C11-GAL4, rh6*^{1}*/rh6*^{1} (gray, n=45), and *UAS-dTrpA1/+; rh6*^{1}*rh6*^{1} (gray, n=49) flies. Mean ± 95% confidence interval is shown. One-way ANOVA with Bonferroni post hoc test.

bioRxiv preprint doi: https://doi.org/10.1101/2023.08.28.555147; this version posted August 29, 2023. The copyright holder for this preprint (which was not certified by peer review) is the author/funder, who has granted bioRxiv a license to display the preprint in perpetuity. It is made available under aCC-BY 4.0 International license.

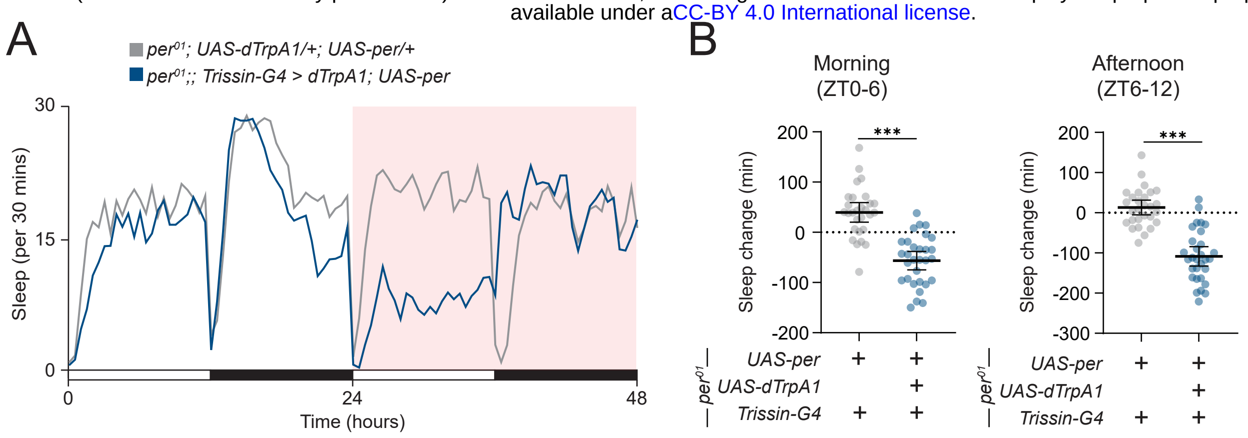

Figure S4

bioRxiv preprint doi: https://doi.org/10.1101/2023.08.28.555147; this version posted August 29, 2023. The copyright holder for this preprint (which was not certified by peer review) is the author/funder, who has granted bioRxiv a license to display the preprint in perpetuity. It is made available under aCC-BY 4.0 International license.

Figure S4 – Rescue of *per* expression in E1 neurons is not sufficient to drive rhythmic output, Related to Figure 5

(A) E1 neuron 24 hr thermogenetic activation in *per* mutants with E1 *per* rescue. Sleep profile for 48 hrs of per^{01}*;;Trissin-GAL4>UAS-dTrpA1; UAS-*per^{16} (blue) and per^{01}*;;Trissin-GAL4>UAS-*per^{16} (gray) flies in 12:12 LD plotted in 30 min bins. Red background indicates increased temperature (28°C), compared to 22°C baseline.

(B) Daytime sleep change with *per* mutant E1 neuron activation and E1 *per* rescue. Sleep change in the morning (left, ZT0-6) and afternoon (right, ZT6-12) compared to baseline day for per^{01}*;;Trissin-GAL4>UAS-dTrpA1; UAS-*per^{16} (blue, n=30) and per^{01}*;;Trissin-GAL4>UAS-*per^{16} (gray, n=28) flies. Mean ± 95% confidence interval is shown. One-way ANOVA with Bonferroni post hoc test.
